# Optical remote sensing for monitoring flying mosquitoes, gender identification and discussion on species identification

**DOI:** 10.1007/s00340-018-6917-x

**Published:** 2018-02-17

**Authors:** Adrien P. Genoud, Roman Basistyy, Gregory M. Williams, Benjamin P. Thomas

**Affiliations:** 1 Department of Physics, New Jersey Institute of Technology, 323 Martin Luther King Jr Blvd, Newark, NJ, USA; 2 Center for Vector Biology, Rutgers University, 180 Jones Ave., New Brunswick, NJ, USA

## Abstract

Mosquito-borne diseases are a major challenge for Human health as they affect nearly 700 million people every year and result in over 1 million deaths. Reliable information on the evolution of population and spatial distribution of key insects species is of major importance in the development of eco-epidemiologic models. This paper reports on the remote characterization of flying mosquitoes using a continuous-wave infrared optical remote sensing system. The system is setup in a controlled environment to mimic long-range lidars, mosquitoes are free flying at a distance of ~ 4 m from the collecting optics. The wing beat frequency is retrieved from the backscattered light from mosquitoes transiting through the laser beam. A total of 427 transit signals have been recorded from three mosquito species, males and females. Since the mosquito species and gender are known a priori, we investigate the use of wing beat frequency as the sole predictor variable for two Bayesian classifications: gender alone (two classes) and species/gender (six classes). The gender of each mosquito is retrieved with a 96.5% accuracy while the species/gender of mosquitoes is retrieved with a 62.3% accuracy. Known to be an efficient mean to identify insect family, we discuss the limitations of using wing beat frequency alone to identify insect species.

## 1 Introduction

Mosquito-borne diseases are a major challenge for human health as they affect nearly 700 million people every year and result in over 1 million deaths [1–4]. Vector control strategies, such as environmental, chemical and biological controls, remain the most effective ways to tackle this issue for multiple reasons: (1) many diseases such as dengue fever, West Nile virus, or Zika virus still have no effective cure; (2) when vaccines or effective treatments exist, they may remain unavailable or unaffordable to large population groups; (3) even in developed countries where populations have access to high quality healthcare, the disease and/or treatments may still cause major nuisances and discomfort.

To be efficient, vector control strategies require accurate data on the fine-scale spatial distribution of each mosquito species. Over the past decade, advances in geographic information system technology have facilitated the development of predictive spatial models for risk of exposure to key vectors [5–8]. However, as stressed by Eisen et al. [9], lack of reliable data on the spatial distribution of key vectors has become a major limitation in the development of spatial epidemiologic and eco-epidemiologic models. The National Health Security Strategy and Implementation Plan 2015–2018 [10] underlined that, with global warming, tropical and sub-tropical species can potentially reach new habitats and severely change the distribution of disease vectors. Similarly, the World Health Organization in its Global Strategy for Dengue Prevention [11] states that “surveillance is a critical component of any dengue prevention and control program because it provides the information necessary for risk assessment and program guidance, including epidemic response and program evaluation”.

Current methods to monitor mosquito populations are very limited. They rely mostly on physical traps using light, pheromones, food, or CO_2_ as bait [12, 13]. Traps allow for a very extensive study of the captured specimens with almost 100% identification accuracy and have been successfully used in a great number of studies. However, they can be tedious to set up (in trees, wetland, difficult topographies), require long laboratory analysis by qualified personnel where each insect has to be counted and identified [14, 15], and have many biases regarding species, age, and sex groups [16–18]. Furthermore, current traps provide a very poor estimate of actual population size because the attractive range of the traps is generally unknown. Without an accurate estimate of population density, it is difficult to determine action thresholds, evaluate insecticide performance, or calculate disease risk [19]. Another approach to monitor mosquitoes is based on hyperspectral imagery, from satellites such as Landsat or NOAA’s polar-orbiting satellites [20–25], or from aircrafts [26], where breeding sites or potential habitats are derived from measuring green vegetation or water normalized index. This methodology offers valuable data on a much larger scale, but provides only indirect data; it cannot evaluate the actual mosquito population, nor confirm the presence of specific mosquito species in a sensitive area. Interesting research using radars has been conducted to study insects and birds [27–30], however most insects are smaller than radio waves, and therefore the use of large wavelengths often limits the size of observable insects. Alternatively, laboratory studies using acoustic or optical instruments demonstrated that the wing beat frequency can be used to identify insects [31, 32, 59, 61]. However, acoustic measurements are limited to very short range due to the low intensity of the acoustic signal. On the other hand, optical measurements are generally performed in a light transmission configuration, where the insect is placed between a light source and a light detector. This configuration is well-suited for laboratory measurements, but limited for field measurements: the light source and detector need to be placed at both ends of the optical path, which makes the instrument stationary.

Thus, developing a reliable technique of remote sensing capable of characterizing insects over large distance is called for. We argue that a viable alternative to the existing methods lies in optical remote sensing technologies such as lidars (light detection and ranging [33, 34]). This remote sensing methodology is widely used for studying atmospheric processes, such as aerosols concentration and formation [35–40], measurements of spatio-temporal distributions of trace gases such as, among others, CO_2_ [41], CH_4_ [42, 43], O_3_ [44], H_2_O [45, 46], and also of volatile organic compounds [47]. Recently, studies have made use of lidar to study entomology [48–56] where large insects (moths, dragonflies, honey bees) are observed and differentiated using the wing beat frequency. While the actual genus and species remain unknown, these measurements provide valuable data on the population dynamics. These methodologies also offer a much higher time resolution, where insect’s activity can be monitored in real-time, enabling behavioral studies during short-time events such as rain, dusk and dawn [56]. Fluorescent measurements [50] allows for a spatially and temporarily resolved measurements, although insects need first to be captured and covered with fluorescent dust prior to being released. As insects transit through the laser beam, the orientation of the wings rapidly changes, producing amplitude modulations in the backscattered signal. Thus, the wing beat frequency can be retrieved by applying a Fourier transform on the recorded signal. The wing beat frequency is known to be an efficient and reliable mean to identify insect family, and to a lesser extent, the insect genus [57–59]. Wing beat frequencies tend to vary significantly between insect families, from a few Hz (such as butterflies) to 1 kHz (such as mosquitoes or biting midges), with little difference between individuals from a same family. In the case of mosquitoes, the flight tone is characteristic of gender, where males present a higher wing beat frequency than females [59, 60].

In this contribution, we present a laboratory study where an infrared optical remote sensing system is used to remotely characterize mosquitoes transiting through the laser beam. The optical system is based on a continuous-wave (CW) laser operating at 1320 nm wavelength with an average power of approximately 3.6 W and a co-axial parabolic mirror to collect the backscattered light. In comparison with the aforementioned lidar publication [48–56], the present work focuses on laboratory measurements to collect data on insects prior to field measurements. As such, we expect the identification accuracy to be improved without the need to capture insects during field measurement campaigns.

Mosquitoes are flying freely in a Plexiglas tube at a range between 3 and 4.25 m from the laser source and collecting optics. The system is designed so that field measurements at a larger range could be achieved by simply increasing the size of the collecting optics, using a Newtonian telescope for example. Both genders of three mosquito species have been considered in this work: *Aedes albopictus, Aedes aegypti*, and an unknown species of the C*ulex* genus. For the first time to our knowledge, backscattered signals from flying mosquitoes are measured, from which the wing beat frequency is retrieved. This contribution focuses on assessing wing beat frequency as a predictor variable to differentiate and identify mosquito species and gender.

For mosquitoes, the wing beat frequency ranges from 100 to 1000 Hz [48, 59–61]. The wing beat frequency is known to be an efficient and reliable means to differentiate insects [57–59]. In addition, male and female mosquitoes are known to have different wing beat frequencies [59, 60]. For example, from our experimental results on *Ae. albopictus*, males have an average wing beat frequency of 681 Hz while females have one of 456 Hz. Mosquitoes sometimes modulate their flight tone to communicate, for some species, male and female flight harmonics converge toward a common frequency while mating [59, 62]. However, these events are rather rare making wing beat frequency a reliable mean to differentiate male from female mosquitoes. In addition, the wing beat frequency is a function of the atmospheric conditions, mainly the temperature [61]. While all measurements presented here have been achieved at a constant temperature (18 ± 1 °C), corrections could be applied in the case of a change in temperature using previous contributions [61].

From transit signals, each insect is characterized using a Naïve Bayes classifier [63, 64]. This classifier was chosen for its implementation simplicity and good performance with independent variables [65]. Two different scenarios were considered: first, a gender classification is presented where male and female of all considered species are discriminated using the sole wing beat frequency as a predictor variable. Then, a similar methodology using 6 classes is applied to identify the species and gender of the transiting insect. As it is a laboratory study, mosquitoes’ gender and species are known a priori in a controlled environment. Therefore, we evaluate the overall accuracy of both scenarios and discuss our results.

The paper is organized as follows. First the methodology to retrieve the optical signals and wing beat frequency is described together with the classification methodology used in this work. Then, experimental results are presented where one typical transit signal is shown and statistics over 427 transit signals are displayed. Finally, we discuss, based on our experimental results, the relevance of the wing beat frequency as a predictor variable for mosquito gender and species identification.

## 2 Methodology

### 2.1 Experimental method

**Fig. 1:**
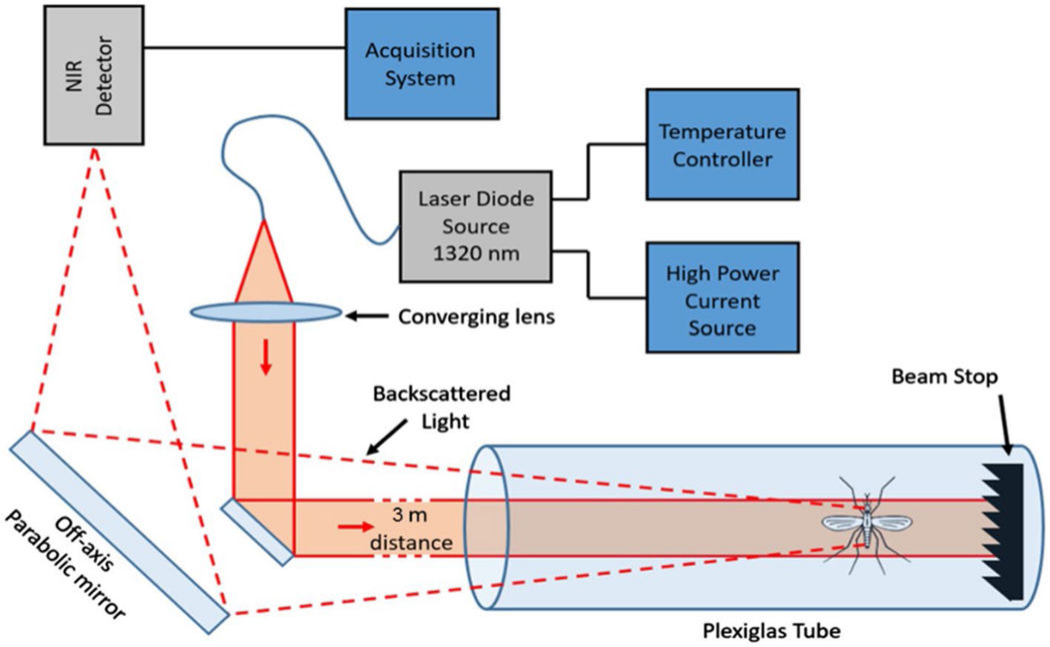
Optical layout of the infrared optical sensor

In this section, the method to measure the optical signal and retrieve the wing beat frequency is explained. Figure 1 presents the optical layout of the system. A CW infrared laser diode source emitting at 1320 nm wavelength and 3.6 W power is collimated so that the Gaussian beam reaches 2 cm FWHM. The laser source is a CW source as it allows to continuously monitor insects, thus avoiding dead times that would cause a pulsed laser. The 1320 nm wavelength of the laser has been adequately chosen to ensure a measurable backscattering power while remaining outside of the visual perception of the mosquitoes as different wavelength are known to influence the Culex Quinquefasciatus mosquito [66]. The electro-retinogram of the female *Aedes aegypti* mosquito showed no response for wavelength above 700 nm [67]. However, a later study demonstrates an influence on flight pattern for lights in the near infrared for the *Anopheles gambiae* mosquitoes [68]. Nonetheless the same study also showed no significant light sensitivity for wavelength above 900 nm. The wavelength dependency for the absorbance of *Anopheles gambiae* and *Anopheles arabiensis* was measured in Mayagaya et al. [69]. This study allows for an educated guess on the range of wavelength at which a non-negligible backscattering of the laser beam by the mosquitoes can be expected.

The beam is transmitted through a Plexiglas tube (1.25 m long, 12.5 cm diameter) located 3 m away from the output mirror. The beam enters the tube through a borosilicate glass tilted 5° downward at one end of the tube, and is stopped by an IR beam-stop at the other end. Mosquitoes are introduced in the tube through small apertures. A 7 cm diameter off-axis parabolic gold mirror focuses the light through the 9.8 mm aperture and on the 3.14 mm^2^ active area of an InGaAs amplified photodetector with a 67 kHz DC bandwidth to collect the backscattered light from the mosquitoes. The laser beam and parabolic gold mirror are set to be coaxial. Signals are recorded using a 16 bit 250 MS/s 125 MHz bandwidth digitizer.

**Fig. 2:**
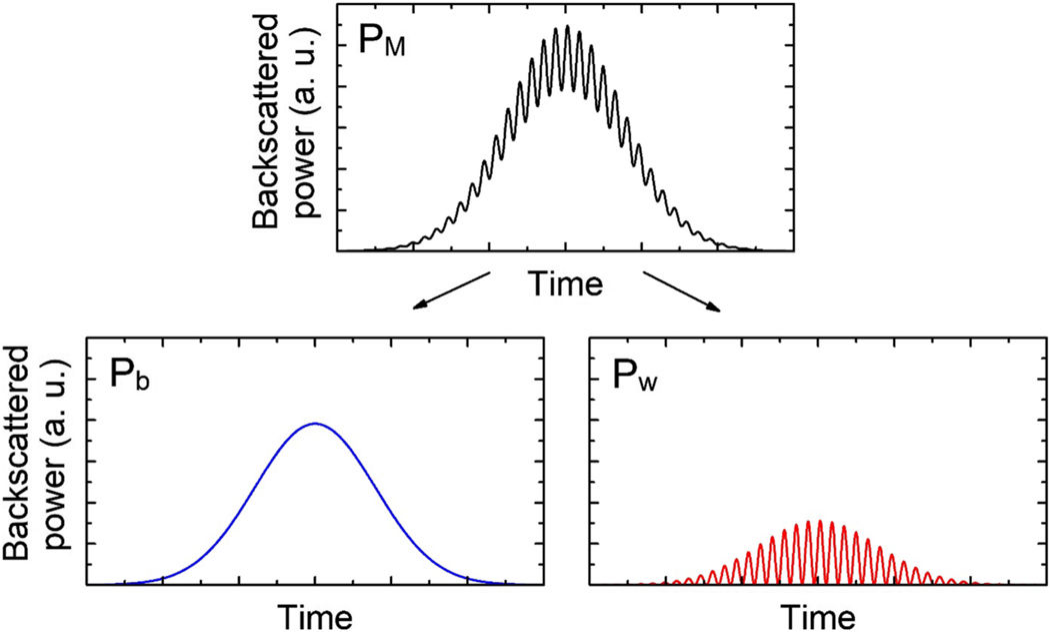
Representation of the total backscattered power (top) and body and wing backscattered power (resp. bottom left and bottom right)

The Eq. 1 below presents the general expression for the backscattered intensity from an insect at a distance *d*.

(1) $$I=\frac{K}{d^{2}}\cdot I_{0}\cdot \beta +I_{B},$$

where *β* is the backscattering coefficient of the mosquito expressed in sr^−1^, describing the incident light backscattered by the insect. *K* is a constant taking into account the size of the off-axis parabolic mirror, quantum efficiency of the detector, and optical transmission or reflection coefficients of the transmitting and collecting optics. *I*_0_ is the initial intensity of the laser beam while *I*_B_ is the light intensity received by the detector from either scattering on the borosilicate glass, backscattering from gas molecules or particles in the probed volume of air, or scattering from the beam stopper terminating the optical path. The optical transmission of the air is considered to be negligible over such distance. The contribution of *I*_B_ to the recorded signal *I* can be treated as constant, especially over such short period of time since the average transit time of a mosquito through the laser beam is 127 ms. Therefore, this contribution can then be compensated by subtracting the average value of the background to the raw data. The optical signal is proportional to the backscattered power by the mosquito *P*_M_ within the field of view of the telescope. The backscattered power from the wings *P*_w_ and body *P*_b_ of the insect are additive since the optical power measured by a detector can, within reasonable range of power, be considered additive. Therefore, *P*_M_ is equal to the sum of the backscattered power within the field of view of the telescope from the wings and body, i.e., *P*_M_ = *P*_b_ + *P*_w_. Both *P*_b_ and *P*_w_ are proportional to the backscattering cross-section of the mosquito which is a function of the projected-area of the body and wings of the insect, respectively (area projected on the telescope plan), as well as the body or wings scattering efficiency. The body projected-area is rather constant during the insect transit. Thus, as the mosquito crosses the beam, the contribution of the body to the optical signal follows a Gaussian shape due to the Gaussian spatial profile of the laser beam. The wing backscattered power *P*_w_ varies with the wing’s position, going from a maximum when the wing plan is normal to the laser optical axis (maximum projected-area), and a minimum when the wing plan is parallel to the laser optical axis (minimum projected-area). Therefore, the body and wing contributions can easily be separated. Figure 2 displays a schematic representation of the backscattered power *P*_M_ corresponding to an insect transiting through a Gaussian laser beam. Local minimums of *P*_M_ correspond to the time at which the wings have a negligible contribution to the backscattered light, i.e., *P*_w_ = 0, *P*_M_ = *P*_b_. Then, local minimums are interpolated to provide the body backscattered power *P*_b_ over the whole transit, allowing the retrieval of the wing backscattered power *P*_w_ = *P*_M_ − *P*_b_ over the whole transit.

The wing beat frequency of mosquitoes ranges from 100 to 1000 Hz [48, 59–61]. Therefore, each transit, lasting generally around 100 ms, allows for the recording of multiple wing beat cycles. A Fourier transform on *P*_w_(*t*) provides the fundamental wing beat frequency *f*_w_ and harmonics.

Three mosquito species, males and females, were studied in this work:

- *Aedes albopictus*, also known as the Asian tiger mosquito, is an important epidemiological vector for infectious diseases such as yellow fever, dengue fever and Chikungunya fever. It is originally found in tropical and sub-tropical areas of Southeast Asia, though this species has recently spread in Western countries.
- *Aedes aegypti*, largely known to be one of the main vectors of yellow fever, but also dengue fever, and Zika virus. This species originated in Africa, but can now be found in tropical and sub-tropical regions. *Ae. aegypti* is responsible for the Zika virus outbreak in 2015–2016 in Brazil, and several regions of South and North America.
- *Culex Genus (unknown species)* from which several species are vectors of disease such as West Nile virus and multiple forms of encephalitis. *Culex* are widely geographically spread and one of the most encountered mosquito genera in North America.

The *Aedes* mosquitoes were reared by the Department of Entomology of Rutgers University, New Jersey. The *Culex* mosquitoes were collected in the field in Hudson County, New Jersey. All the specimens were studied shortly after they hatched, less than 7 days old. To increase the likeliness of observing a mosquito transiting through the laser beam, they were introduced into the enclosure by batch of ten specimens of the same gender and species. Measurements were performed over the following days until mosquitoes would naturally die, generally around 15–20 days after hatching. The mosquitoes of those three different species measure between 3 and 6 mm and display a sexual dimorphism with female larger than male. Despite their small size the backscattered signals from mosquitoes were sufficiently high to be unequivocally discriminate from the background noise.

### 2.2 Event classification

For each mosquito event recorded, a wing beat frequency *f*_w_ can be determined. From all these frequency measurements, both a mean (*μ*) and standard deviation (*σ*_SD_) are calculated using Eqs. 2 and 3, respectively. For each class, a probability density function is derived from the measurements where mosquito species and gender are known. Those functions are then used as predictor variables to retrieve the likeliness that each measured wing beat frequency will originate from a specific class. Two classifications are discussed in the paper: the first one where only two classes corresponding to the mosquito gender are considered. The second where male and female of the three mosquito species are differentiated resulting in 6 classes.

(2) $$\mu =\frac{\sum_{i=1}^{N}\frac{f_{\text{w}i}}{\sigma_{i}^{2}}}{\sum_{i=1}^{N}\frac{1}{\sigma_{i}^{2}}},$$

(3) $$\sigma_{\text{SD}}=\sqrt{\frac{1}{N-1}\sum_{i=1}^{N}{\left(f_{\text{w}i}-\mu \right)}^{2}},$$

where *N* is the number of events in the evaluated class, *f*_w*i*_ and *σ*_i_ the wing beat frequency and uncertainty respectively. The uncertainty is defined as half the FWHM of the fundamental frequency of the Fourier transform on *P*_w_(*t*). The wing beat frequency mean value μ is a weighted mean, which will give a greater strength to the more accurate values while reducing the influence of the more uncertain ones. This was done in regards of experimental uncertainty that fluctuate from around ± 2 Hz up to ± 50 Hz. This uncertainty is most likely due to a change of wing beat frequency during the time of transit as mosquitoes can slightly modulate their wing beat frequency [59, 62]. For each wing beat frequency *f*_w*i*_, the most likely class *C*_*j*_ will be determined through a Bayesian classifier. The experiment was done in a way that the actual class of each measured event is known. This will allow for the evaluation of the accuracy of the Bayesian classifier with regards to species and/or gender. The probability that a measurement *f*_w*i*_ belong to the class *C*_*j*_, *P*(*C*_*j*_|*f*_w*i*_), is described as follows [63].

(4) $$P\left(C_{j}|f_{\text{w}i}\right)=\frac{P\left(C_{j}\right)P\left(f_{\text{w}i}|C_{j}\right)}{P\left(f_{\text{w}i}\right)},$$

where *P*(*C*_j_) is the prior probability of the class *C*_*j*_, *P*(*f*_w*i*_|*C*_*j*_) is the probability of obtaining the value *f*_w*i*_ in the class *C*_*j*_ and *P*(*f*_w*i*_) is the prior probability of the observed frequency *f*_w*i*_. The prior probability, *P*(*C*_*j*_), will be chosen as equal for all classes, like it is often the case for field measurements where it is difficult to know how likely one class is in comparison to another without influencing the obtained results toward the same results as the assumption. The wing beat frequency of mosquitoes from the same species and gender follows a Gaussian distribution [14, 48, 70]. Therefore, *P*(*f*_w*i*_|*C*_*j*_) can be evaluated using a Gaussian probability function also called probability density function, Eq. 5.

(5) $$P\left(f_{\text{w}i}|C_{j}\right)=\frac{1}{\sqrt{2\pi }\sigma_{C_{j}}}e^{-\frac{{\left(f_{\text{w}i}-\mu C_{j}\right)}^{2}}{2\sigma_{C_{j}^{2}}}},$$

where *σ*_*Cj*_ and *μ*_*Cj*_ are, respectively, the standard deviation and mean value of the class *C*_*j*_. For every class a score, *S*_*cj*_(*f*_w*i*_), defined by Eq. 6 is attributed. This score can be considered as a normalized probability that f_wi_ belong to the class *C*_*j*_.

(6) $$S_{cj}\left(f_{\text{w}i}\right)=\frac{P\left(C_{j}\right)P\left(f_{\text{w}i}|C_{j}\right)}{\sum_{C_{k}\in C}P\left(C_{k}\right)P\left(f_{\text{wi}}|C_{k}\right)}.$$

The score is defined in a way that for each frequency *f*_w*i*_ the sum of the scores for all possible classes is equal to 1. In this regard, the score can be seen as the relative probability of the class *C*_*j*_ in comparison with all other possible classes. Likewise, the ratio of two scores allows for the evaluation of the relative likeliness of one class in comparison to the other.

The attributed class of any measurement *f*_w*i*_: *A*_*Cj*_(*f*_w*i*_) will be the class *C*_*j*_ for which *S*_*cj*_(*f*_w*i*_) is the greatest, since it is statistically the most likely, but it is not necessarily the correct one.

(7) $$A_{Cj}\left(f_{\text{w}i}\right)=\text{arg}\;{\text{max}}_{c_{j}\in C}\left(S_{cj}\left(f_{\text{w}i}\right)\right).$$

Once the Bayesian classification has been applied, the accuracy for each predicted class can be calculated. Data presented in this paper are obtained in a controlled environment, thus the actual class of every measurement is known and the veracity of every class prediction can be easily evaluated. Hence, the class accuracy, CA_*j*_, of the class *j* can be defined using Eq. 8.

(8) $${\text{CA}}_{j}=\frac{N_{j}}{N_{j}+N_{i\neq j}},$$

where *N*_*j*_ is the number of correctly predicted events as class *j* and *N*_*i*≠*j*_ the number of events wrongfully predicted as class *j*. The accuracy of every predicted class can be determined and therefore the overall accuracy of the entire classification (OAC) can be evaluated using Eq. 9.

(9) $$\text{OAC}=\frac{\sum N_{j}}{\sum N_{j}+\sum N_{i\neq j}},$$

where ∑*N*_*j*_ is the sum of all the correct predictions and ∑*N*_*i*≠*j*_ the sum of all the wrong ones.

## 3 Experimental results and discussion

### 3.1 Data analysis and results

**Fig. 3:**
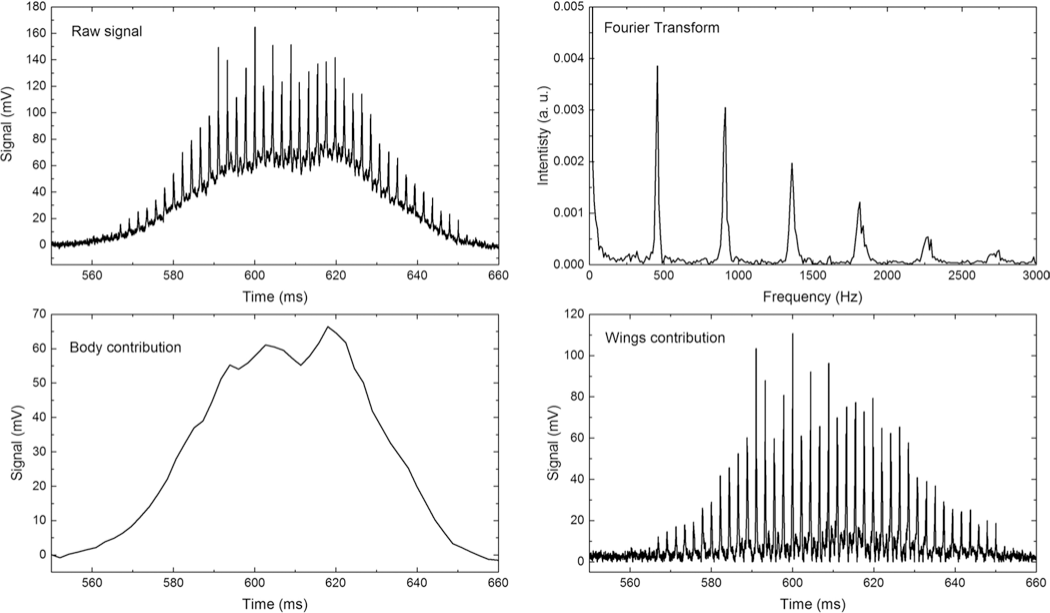
Raw signal of the mosquito transiting through the laser beam (top left) together with the body and wings contribution (resp. bottom left and bottom right). The Fourier transform of the wing contribution provides with the fundamental wing beat frequency and harmonics of the insect (top right)

First, this section presents an example of an optical signal showing the transit of a mosquito through the laser beam, the retrieval of its body and wing backscattering coefficients and the Fourier transform leading to the wing beat frequency and harmonics. Then, statistics over 427 transit signals are presented showing the frequency distribution for each gender and species. Figure 3 displays the raw signal measured when a mosquito transits through the laser beam (top left). Raw signals are characterized by their Gaussian shape due to the spatial profile of the beam and sharp intensity peaks due to the wings’ orientation rapidly changing. As a result of the insect sometimes changing directions or hovering while crossing the beam, transit times varied from a few ms up to 1300 ms with an average of 127 ms. The body and wings contributions to the raw signal (resp. bottom left and bottom right) are differentiated using the methodology described in Sect. 2.1. Finally, the Fourier transform of the wings contribution (top right) enables the measurement of the fundamental mosquito wing beat frequency and harmonics. The relative strength of the odds and evens harmonics is related to the orientation of the insects at the time of the measurement [54]. The ratio between the intensity of the second over the first harmonic may allow the differentiation between an observation from the front or back of the mosquito and one from the side. This is furthermore supported by the diptera insect model presented in Brydegaard [53]. This model predicts that the intensity of the second harmonic will be higher than the intensity of the first if the insect is observed from the side as the apparent surface of the wings will appear large twice during one wing beat cycle. Still, we found this model unsuited to the specific wing pattern of the mosquito as they display unusual wing movement in comparison with most other flying insects [71].

**Fig. 4:**
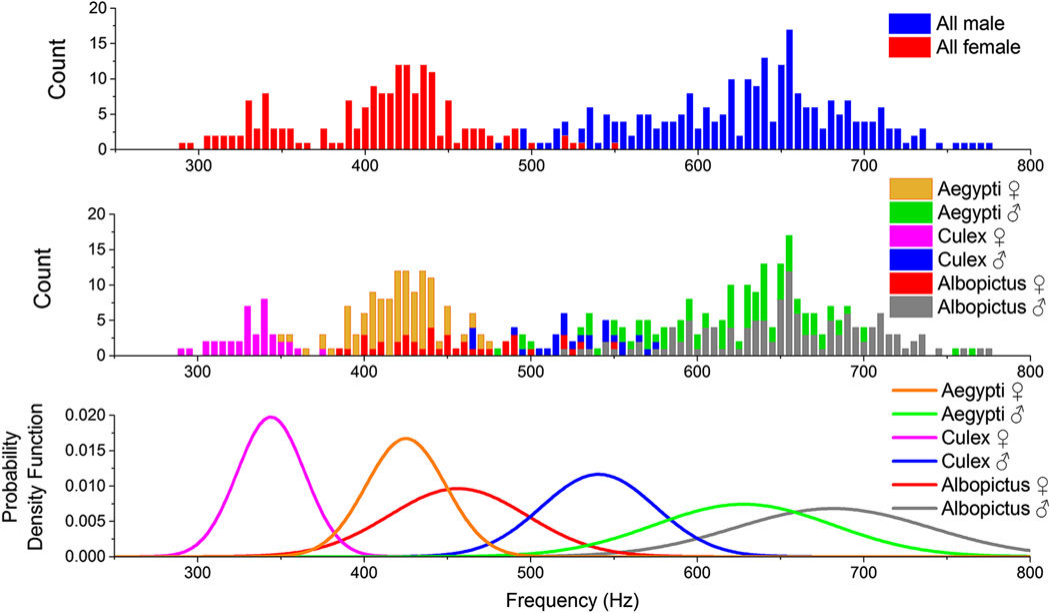
Histogram of the measured wing beat frequencies for all females and males (top), all species and both genders (middle) and their respective probability density function (bottom). Every bar has a width of 5 Hz and its value is the number of events measured within this frequency window

This analysis of raw signals was applied to all recorded transit signals. In the end, 427 mosquito transit events, spread between the 3 species for both genders were recorded. All the retrieved wing beat frequencies are between 200 and 900 Hz as expected for mosquitoes [48, 59–61]. As several mosquitos are simultaneously present into the enclosure, there is a possibility that two or more specimens would cross the laser beam at the same time. Although this particular issue did not occur in any of the 427 measurements, the possibility to flag such event must be addressed. This phenomenon has not been extensively studied, but in such case, the Fourier Transform is expected to display two or more fundamental frequency peaks which should be enough to flag the event. Sill this question remain open and further investigation will be conducted. Figure 4 displays the 427 events on two histograms, top graph with all female and male and middle graph when separated into 6 classes, corresponding to both genders of the 3 species. The bottom graph displays the probability density functions for the six classes. These results are discussed in Sect. 3.2 and 3.3.

From all these measurements, an average wing beat frequency and standard deviation is determined for each class and presented in Table 1. To evaluate the uncertainty, for the 68% confidence interval (1 *σ*), on the average wing beat frequency of the different classes, Eq. 10 can be used to determine the standard error σ_SE_.

(10) $$\sigma_{\text{SE}}=\frac{\sigma_{\text{SD}}}{\sqrt{N}},$$

where *σ*_SD_ is the standard deviation and *N* the number of measurements in the class.

### 3.2 Classification results

In this section, we discuss the accuracy when distinguishing male from female mosquitoes using data from the infrared optical system with a Naïve Bayes classifier. Then, a similar approach is presented to evaluate species/gender classification.

The only feature used as predictor variable in this classification is the wing beat frequency. Every class has a distinctive average wing beat frequency, which supports that the wing beat frequency can be used as a discriminatory factor between classes. Considering the standard deviation of the laboratory gathered data, some overlap between classes is unavoidable and will be the restricting factor of the discrimination attempt. As previously described in Sect. 2.2, a Bayesian classifier, solely based on the wing beat frequency, was applied using the data regrouped in Table 1. When applied to the gender classification, the score for the male class is equal to a third of the sum of the score for male *Ae. albopictus*, male *Culex* and male *Ae. aegypti* classes and similarly for the female class. The confusion matrix for the two classifications are presented in Tables 2 and 3. For both classifications, the class accuracy gives an insight into the efficiency of the wing beat frequency alone as a discriminatory factor between gender and species/gender of studied mosquitoes.

For the gender classification, the wing beat frequency alone allows for an accuracy of 95.8 and 97.6% for the male and female class respectively and an overall accuracy of 96.5%. The same Bayesian classifier as for the gender classes is then applied to a more complex classification, for both species and gender. This new classification now contains six different classes. For this classification, the class accuracy displays disparate values ranging from 36.2 to 88.6%. The overall accuracy of this classification is 62.3% which results from larger overlaps between the probability density functions due to the increasing number of classes.

### 3.3 Discussion

Wing beat frequency is known as an efficient mean to differentiate gender from previous studies using either microphones or optical instruments. The results obtained in this study confirmed that this approach remains appropriate when using an infrared CW optical remote sensing system based on backscattered light. Considering the relative simplicity of the classifier and the unicity of the discriminatory factor, this result demonstrates the effectiveness of the wing beat frequency for gender discrimination of mosquitoes. This is a promising result since the gender differentiation is paramount to the efficiency of the sterile insect technique, which is a cost effective mitigation technique employed to reduce insect populations such as mosquitoes [72–74].

For the gender/species classification, the overall accuracy of the classification drops down to 62.3%. While three of the six classes still have an accuracy above 74% (female *Culex*, female *Ae. aegypti* and male Ae. *albopictus*), others drop to a rather low accuracy, down to 36.2%. The less accurate class still has more than twice the accuracy of a random prediction, which proves that wing beat frequency is a valuable predictive variable for classification methods for species identification. However, we believe that wing beat frequency alone is insufficient to properly classify mosquito species. Some of the class accuracy retrieved for species/gender identification in the work presented here are below 40%, despite considering only three species. When used for field experiments, more potential mosquito species need to be considered, leading to more classes and larger overlaps between each wing beat frequency distribution, resulting in lower class accuracies. As for example, New Jersey counts a total of 63 potential mosquito species, Florida up to 80. Therefore, the sole wing beat frequency may be used as predictor variable only in the case where only a few species might be encountered or in the case where only the insect family is retrieved (such as discriminating butterflies, flies or moths from mosquitoes). However, in the case of field measurements where dozens of mosquito genera and species may be present, wing beat frequency alone will not be sufficient to identify them with good accuracy.

A common way to improve the class accuracy would be to add other predictive variables. By recording the time and place where field measurements are performed, data on the circadian rhythm of flight activity together with the spatial distribution of the mosquito species can potentially lead to better class accuracy [70]. While we acknowledge that different species of mosquitoes have different activity times (dawn, dusk, day or night) and can be found on different parts of the globe and habitats, we argue that these additional predictive variables may be counterproductive and that, at the very least, should be handled with care. For instance, the circadian rhythm cannot be used without at least a rough estimate of the population of each considered species. If a species A with a small population has an activity peak while another species B with a much larger population has a low activity, their probability to interact with the instrument may be equal, although in this case, the classification will consider species A to be much more likely, which will induce a strong bias in the results.

In addition, with the accuracy decreasing with the number of considered classes, the weight of the wing beat frequency as predictive variable in the classification methodology is reduced when compared to other predictive variables: assumptions made on the circadian rhythm and spatial distribution become predominant in the classification process, especially if the wing beat frequency is weighted based on its uncertainty. This would lead to an inherent problem for entomological instruments specifically developed to study the spatial distribution and activity of insects. Actual changes in the behavior of mosquitoes could go unnoticed. For entomological lidars or optical remote sensing system using a classification methodology to retrieve information, this specific information cannot be used as a predictive variable or it would otherwise skew the results towards the current state of knowledge. For instance, using available data on the spatial distribution of mosquito species as a predictive variable to monitor the migration of a mosquito, such as the spread of tropical species over larger latitudes with climate change would be biased. Similarly, using circadian rhythm as a predictive variable to study the impact of pesticide would lead to biased results as it may affect species differently.

## 4 Conclusion and outlooks

In this contribution, we present an infrared continuous-wave optical sensing system to remotely study flying mosquitoes in real-time. The wing beat frequency of each insect transiting through the infrared laser beam is retrieved with 1.9% error using a Fourier Transform of the amplitude modulation of the backscattered light. The system is used in laboratory conditions, where male and female mosquitoes of three different species, *Ae. albopictus, Ae. aegypti*, and an unknown species of the *Culex* genus, are known a priori and studied from a distance between 3 and 4.25 m. A total of 427 transit signals have been recorded, providing enough data to evaluate the performance of a Bayesian classification method to identify, at first, only the mosquito gender, and then, the gender and species. In the case of gender identification, results show that male and female are discriminated with an overall accuracy of 96.5% despite the broad range of possible frequency of male and female mosquitoes of different species. When attempting to discriminate both gender and species, the overall accuracy of the classification drops to 62.3%, with class accuracy ranging from 36.2% for male *Culex* to 88.6% for female *Culex*. While the overall accuracy of 62.3% could be seen as acceptable, the experiment here only considered both genders of 3 species. In the case of field measurements, the number of species at one location is generally in the order of a dozen or more. It is likely that the overall accuracy will decrease significantly if more species are considered. These results demonstrate that wing beat frequency is a sufficient predictive variable to identify the gender of a mosquito, though the sole use of wing beat frequency to identify mosquito species is insufficient. Hence, when applied for species identification, wing beat frequency needs to be used with other independent predictive variables, such as optical properties of the observed insect, to provide accurate and un-biased mosquito species identification. Examples of such optical properties are given by Gebru et al. [54] where the ratio of the optical cross-section of honey bees in the near and shortwave infrared is measured. Similarly, Shaw et al. [75] measured the depolarization ratio of honey bees using a Lidar. Additionally, other predictor variables could be investigated, such as the shape of the periodic function of the wing contribution, the ratio of intensities between body and wing contribution, or transit times. Classification using deep machine learning technics may be well-suited to take these numerous variables into consideration.

## Figures and Tables

**Table 1:** Average wing beat frequency and uncertainty for both gender of the *Ae. albopictus, Ae. aegypti* and *Culex* mosquitoes

	Male			Female		
Average wing beat frequency (Hz)	617 ± 3			408 ± 5		
Standard deviation (Hz)	52			64		
	*Ae. albopictus*	*Culex*	*Ae. aegypti*	*Ae. albopictus*	*Culex*	*Ae. aegypti*
Average wing beat frequency (Hz)	681 ± 5	541 ± 7	628 ± 6	456 ± 6	344 ± 3	425 ± 2
Standard deviation (Hz)	59	34	54	41	20	24

**Table 2:** This table display the actual class of every event versus their predicted class by the Bayesian classifier, also known as confusion matrix, data separated by gender

Actual	Predicted	
	Male	Female
Male	248	4
Female	11	164
Class accuracy (%)	95.8	97.6

**Table 3:** Confusion matrix and class accuracy for the Bayesian classifier, data separated by gender and species

Actual	Predicted					
	*Ae. albopictus*		*Ae. aegypti*		*Culex*	
	Male	Female	Male	Female	Male	Female
*Ae. albopictus*						
Male	61	0	60	0	11	0
Female	0	14	0	21	6	0
*Ae. aegypti*						
Male	21	3	50	0	20	0
Female	0	9	0	81	0	5
*Culex*						
Male	0	5	0	0	21	0
Female	0	0	0	0	0	39
Class accuracy (%)	74.4	45.2	45.5	79.4	36.2	88.6
